# Exploring the Risk of Resulting in Homicide and Suicide in Spanish Missing Person Cases

**DOI:** 10.1007/s10610-023-09536-y

**Published:** 2023-03-13

**Authors:** Néstor García-Barceló, Miguel Ángel Alcázar Córcoles, Javier Revuelta Menéndez, Penny Woolnough, José Luis González Álvarez

**Affiliations:** 1 Department of Biological and Health Psychology, School of Psychology, Autonomous University of Madrid (UAM), Madrid, Spain; 2 Institute for Forensic and Security Sciences (ICFS), Autonomous University of Madrid (UAM), Francisco Tomás Y Valiente Street, Madrid, Spain; 3 Department of Social Psychology and Methodology, School of Psychology, Autonomous University of Madrid (UAM), Madrid, Spain; 4 Division of Psychology, School of Health and Social Sciences, Abertay University, Dundee, Scotland; 5 Ministry of Interior, Spanish Secretary of State for Security, Madrid, Spain

**Keywords:** Homicide, Suicide, Missing persons, Risk assessment, Risk factors, Prevention

## Abstract

The study explores in depth the relationship between missing persons’ psychosocial and criminological characteristics/circumstances and violent-fatal outcomes (suicide and homicide). A relational analytical explicative study of 929 cases and controls was designed using a retrospective and stratified design. Data gathering was conducted through the content analysis of judicial and police information, as well as the development of psychological autopsy techniques and semi-structured interviews with the persons involved in the missing person cases including offenders in prison. Bivariate and multivariate statistical techniques were utilised for analyses. The findings showed that there are different risk and protective factors which can distinguish between good state of health, suicide, and homicide outcomes. This research entails implications for prevention and police risk assessment system.

## Introduction

Thousands of missing person reports are received each year by law enforcement agencies globally, who have legislated responsibility for investigating them (Ferguson & Soave, 2020; Gong et al., 2017; National Crime Agency, 2016; National Crime Information Center-FBI, 2017; Spanish Ministry of Interior, 2021; Stevenson & Thomas, 2018; Todorović & Butorac, 2017). Although most missing people are traced in a good state of health (GSH), around 3% are located deceased due to an accident, natural causes, homicide, or suicide (Eales, 2017; Foy, 2006; Newiss, 2004; Spanish Ministry of Interior, 2021; Sveticic et al., 2012; Tarling & Burrows, 2004; Woolnough et al., 2019). Identifying the small number of fatal cases from the large number of overall cases is one of the biggest challenges that law enforcement faces daily (Newiss, 2004).

The United Nations Office on Drugs and Crime (UNODC, 2019) defines homicide as any “unlawful death inflicted upon a person with the intent to cause death or serious injury”, whereas suicide is defined by the World Health Organization (WHO, 2014) as “the act of deliberately killing oneself”. In contrast, defining a missing person is complicated and multifaceted, with institutions and authors all over the world using different conceptualisations of the phenomenon (Payne, 1995; Taylor et al., 2018). Despite this variation, most countries’ definition of a missing person speaks to “the challenge of locating a person whose whereabouts are unknown, where the disappearance may be out of character and where they may be the subject of crime or at risk of harm” (Taylor et al., 2018). This highlights the critical need to differentiate those persons who may be at risk of victimisation/harm. However, at an international level, there are no consistent statistics regarding missing person cases which result in homicide or suicide and academic study of missing persons that result in fatal outcomes is much neglected. The small number of existing studies suggests that homicide or suicide outcomes are very low, 0.5% for homicides (Newiss, 2005, 2006) and 2.5% for suicides (Sveticic et al., 2012). Similarly, in Spain, the annual report on missing persons published by the Ministry of Interior (2021) and previous research (García-Barceló et al., 2020a, b, c) indicate that Spanish law enforcement receives between 20,000 and 30,000 reports of missing people every year with 1.1% ending in fatal outcomes (0.15% homicides; 0.42% suicides).

Despite homicide and suicide outcomes having very low base rate outcomes amongst all reported missing persons, such cases generate high concern to public institutions because of the social impact, devastating psychological consequences suffered by families, and the technical difficulties generated for police investigations (Cohen et al., 2008). Specifically, police agencies investigating homicide and suicide cases involving missing persons face unique challenges and barriers, and many investigative failures can be attributed to these problems (García-Barceló et al., 2020c; LePard et al., 2015; Woolnough et al., 2019). Consequently, the empirical identification of risk and protective factors associated with disappearances which result in homicide or suicide will contribute to a greater knowledge about homicide or suicide outcomes, risk scenarios, and the variables which intervene in missing person cases as well as entail different implications both at prevention and police investigation levels (García-Barceló et al., 2019, 2020a, b, c, 2021a; Newiss, 2004, 2005, 2006; LePard et al., 2015; Woolnough et al., 2019). Therefore, identification of risk factors which can distinguish between homicide or suicide outcomes and good state of health (GSH) outcomes will favour the construction of effective and valid risk assessment instruments which will allow the early identification of high-risk and low-risk cases (Eales, 2017; García-Barceló et al., 2021b; Taylor et al., 2018). Despite an absence of research focussed on missing persons ending with homicide or suicide outcomes, over the past two decades, there has been a gradual increase in missing person research focused on the identification of causes and motives for going missing as well as risk factors for harm/fatal outcomes (Biehal et al., 2003; Bonny et al., 2016; Buckley, 2012; García-Barceló, et al., 2019; Huey & Ferguson, 2020). This research agrees that most disappearances do not involve foul play causes, criminal motives, or suicides attempts. In this regard, people may disappear as a result of mental health issues, life pressures, alcohol/drug problems, physical and psychological abuse, negligence or discrimination situations, sexual exploitation, having familial, scholar, emotional or delinquency problems, or just want to be independent (Biehal et al., 2003; Blackmore et al., 2005; Cohen et al., 2008; Foy, 2006, 2016; Gibb & Woolnough, 2007; Greene & Hayden, 2014; Kiernan & Henderson, 2002; Newiss, 2006; Tarling & Burrows, 2004; Payne, 1995, Woolnough & Cunningham, 2020; James et al., 2008; Kiepal et al., 2012; Shalev, 2011; Stevenson & Thomas, 2018; Thompson et al., 2011; Tyler & Cauce, 2002).

Taking a similar approach, some international attempts to identify specific risk factors for fatal outcomes have been accomplished. In this regard, research has identified that those who are male, elderly, in a separation process, a victim of crime, have previous suicide attempts, have alcohol/drug problem, have mental health issues, had suffered an accident, or had committed a crime are related to fatal outcomes (Bantry White & Montgomery, 2015; Bricknell, 2017; Gibb & Woolnough, 2007; Eales, 2017; Newiss, 2004, 2006, 2011; Newiss & Greatbatch, 2019; Sveticic et al., 2012; Tarling & Burrows, 2004). In Spain, only two published studies (García-Barceló et al., 2021a, b) have researched this phenomenon. Firstly, García-Barceló et al. (2021b) used multinomial logistic regression to analyse the relation between general stressors/triggers of 653 adult missing persons and their state of health when located. The findings identified that having emotional problems significantly explained some fatal outcomes such as suicide. On the other hand, considering age as a modulating variable (Alys et al., 2013; García-Barceló et al. (2021a, 2022) conducted a study which sought to identify the main characteristics of 653 missing adults and 487 child cases which resulted in fatal outcomes. This study showed that adults were located deceased in a higher proportion than minors. Also, adults who were located deceased were characterised as being in a separation process, had threatened to take their own life, had suffered an accident, or expressing being depressed, whereas children who were located deceased were characterised by not having a busy lifestyle, not experiencing family disassociation, or family rebellion.

Specifically, although some international research related to foul play in missing person cases is focused on missing abducted or kidnapped children as well as individuals who have experienced abuse or violence, little has been written about missing persons resulting in homicide (Biehal et al., 2003; Bonny et al., 2016; Cohen et al., 2008; Foy, 2006; García-Barceló et al., 2019; James et al., 2008; Salfati & Bateman, 2005). In the UK, Newiss (2004, 2005, 2006) explored the association between some socio-demographics such as gender and age and this kind of fatal outcome in missing person cases. Collectively, the studies identified that the distribution of men of all ages who were missing and suffered a homicide outcome was proportionally lower than the rate for woman of all age groups (1:13,600 vs. 1:4600 respectively). Focusing on children who went missing and suffered a homicide outcome, the distribution of boys between 1 and 4 years old was lower than girls aged between 5 and 9 years old (1:1,800 vs. 1:700). Finally, these studies indicated that victims aged between 14 and 24 years old were more likely to go missing and result in a homicide outcome. Biehal et al. (2003) and James et al. (2008) in Australia also pointed out that adult victims of homicide outcomes abused alcohol and drugs, while for fatal child cases, the parents were in the process of separating. Another point of interest regarding international research in the field of missing person cases is that which has looked at homicide outcomes amongst aboriginal or indigenous woman (Buckley, 2012; Cohen et al., 2008; Kubik & Bourassa, 2016; Savarese, 2017). In Spain, a pilot and descriptive approach focused on an in-depth review of homicide outcomes (Garcia-Barceló et al., 2020c) showed that those persons who went missing because and who were the victim of homicide were mainly characterised by psychosocial and criminological characteristics as well as circumstances which surrounded the disappearance such as having financial, drug, and delinquency problems. They were also characterised by having had a previous argument, procriminal associations, being in a different country from their origin, abusing alcohol/drugs, being in the process of separating, having a busy lifestyle, not being in a familiar place, having financial problems/debts, and previously having been a victim of crime. In contrast, disassociating from one’s family, abandoning medical treatment, and being a repeat missing person did not seem to characterise those resulting in homicide outcomes.

With regard to suicide outcomes, international research has identified that experiencing relationship breakdown/problems, physical health problems, financial problems, having previously attempted suicide, leaving a suicide note, or having mental health issues are related to suicide outcomes (Foy, 2006; Gibb & Woolnough, 2007; Eales, 2017; Sveticic et al., 2012; Woolnough et al., 2019; Yong & Tzani-Pepelasis, 2020). In Spain, there are only two descriptive studies which explore suicides within a sample missing person cases (Larrañaga et al., 2019; García-Barceló et al., 2020a). Larrañaga et al. (2019) described a sample of 41 missing persons who suicided as mostly male and nationals. In a more in-depth study, García-Barceló et al. (2020a) analysed 341 missing person police records using multidimensional scaling and identified an “intentional-dysfunctional” missing persons’ behavioural theme characterised by variables including attempting suicide, having emotional problems, being under the influence alcohol and drugs, having previously verbalized suicidal intent, or left notes.

In summary, the majority of existing missing person research has studied motives and causes of going missing and focused on the exploration of harm and fatal outcomes generally. However, there is very little research focused on the identification of those characteristics which could specifically distinguish between homicide or suicide outcomes and those cases in which the missing person is located in a GSH. Some studies have briefly explored and described socio-demographic factors related to missing person cases which result in homicide outcomes (Buckley, 2012; Cohen et al., 2008; Kubik & Bourassa, 2016; Newiss, 2004, 2006; Savarese, 2017). However, there is a need to go beyond the description promoting robust research allowing the identification of psychosocial and criminological risk factors that may provide an early indication of cases likely to end in homicide or suicide outcomes. In this manner, understanding the complex interplay between key pieces of evidence uncovered during an investigation/search (e.g. a suicide note, mental health issues, leaving a mobile phone behind) could indicate increased likelihood of a suicide, for example, and allow investigators to more effectively and efficiently target resources.

### Purpose of This Study and Research Questions

Given the small amount of research focused on fatal outcomes for missing person cases, as well as the lack of theory which could be used to explain and test why a missing person case results in homicide or suicide, this study aims to explore the risk and protective factors which could influence these outcomes. In this manner, this study attempts to identify whether key distinguishing missing person characteristics and circumstances of the cases are statistically associated with homicide or suicide outcomes. In this manner, it aims to contribute to the generation of new empirical knowledge in the field, identifying risk and protective factors for homicide and suicide, as well as the future development, calibration, and validation of risk assessment tools for homicide and suicide outcomes.

## Method

### Data Collection

Initial data used for this study were extracted from the PDyRH System of CNDES, which has been used by law enforcement in Spain since 2009 to record information about missing persons, cadavers, and unidentified human remains. To identify the basis for a proportionate stratified sample of cases, all cases reported over a ten-year period from 1 January 2009 to 31 December 2019 were initially analysed (*n* = 196,735 cases). After exclusion of unsolved cases (*n* = 14,077; 7.2%), unaccompanied foreign minors (*n* = 39,770; 20.2%), and repeat missing episodes, leaving only the first recorded case for any individual to avoid biasing the data (*n* = 30,120; 15.3%), a total of *n* = 112,768 cases remained. Based on proportionate percentages of territory and police demarcation, the need to analyse a minimum of 10% of the sample to ensure representativeness was identified (*n* = 1127). A decision was taken to over-sample to *n* = 2000 cases based on the inference that some of the reports to be requested would not be received because of the dispersion of the information in local police units. Consequently, CNDES requested 2000 cases, based on proportionate percentages of territory and police demarcation as identified in the initial 10-year sample, from the most recent year (1 January to 31 December 2019). The request was made to Spanish law enforcement, both national (Civil Guard and National Police) and autonomous (Policía Foral de Navarra, Ertzaintza and Mossos d’Esquadra) which are Spanish law enforcement involved in national jurisdictions. The COVID-19 pandemic influenced the celerity of the sample gathering process and 1218 missing persons’ police records were initially received. However, 114 cases had to be rejected because they did not meet the inclusion/exclusion criteria, resulting in a final sample of *n* = 1104 missing person reports.

In order to allow comparison with a sufficient sample of homicide cases, a specific subsample of all police and judicially solved homicides, which began as missing person cases during the previous five-year period (1st January 2014 to 31st December 2018), was also requested. This strategy was based on the fact that approximately 0.15% of missing person cases reported per year relate to homicide, making it necessary to achieve a sample of at least 30 solved cases for analysis. Also, as homicides can be difficult to solve, it was decided that a prior “five-year” sampling period was necessary to guarantee a sufficient subsample. Furthermore, as the wider missing person sample was from 2019, it was deemed necessary to get the most temporally similar sample for homicide cases (i.e. up to 31 December 2018). On this basis, a sample of 36 homicide cases were received taking the overall sample of cases to *n* = 1140. Finally, the dataset was refined to only include those cases in which the missing person was located in a GSH (851; 74.6%) or was located deceased due to homicide (36; 3.2%) or suicide (42; 3.7%). Consequently, harm (182; 16%) and other fatal outcomes (accidental (16; 1.4%), natural (10; 0.9%), and equivocal (3; 0.3%) were excluded. Thus, the final sample comprised 929 missing person reports used in the current study which correspond to 929 missing individuals.

### Participants

The sample comprised 402 (43.3%) females and 527 (56.7%) males; 782 (84.2%) nationals and 147 (15.8%) foreigners; and 461 (49.6%) over 18 year olds (legal age in Spain) and 468 (50.4%) under the age of 18. The age range was from 1 to 86 years old (M = 27.4 years, Mdn = 17 years, SD = 17.1). Regarding outcome, 851 (91.6%) were found in GSH, 36 (3.9%) were homicides, and 42 (4.5%) were suicides.

### Design

For the study of risk factors of homicide and suicide outcomes in missing person cases, a relational analytical-descriptive and explicative study was designed using a retrospective and stratified design. Differences in frequency measures of the analytical cases (homicide) and controls (GSH) were estimated and models which better explain homicide and suicide outcomes were identified.

### Coding and Instruments

A multidisciplinary working group, comprising academics from the disciplines of psychology, criminology, and sociology, and police officers from CNDES (Spanish National Centre for Missing Persons) conducted a review of international research and police investigation protocols for missing persons and developed a coding matrix/dictionary according to the data available from the literature review and police officers’ experience. Following this, content analysis (Andréu, 2002) of the 929 missing person cases reported to police was conducted.

As the data used in this study were not initially gathered for the purpose of scientific research, there was a lack of psychosocial information in the police records. To overcome this limitation complementary to the content analysis, the working group elaborated a template for semi-structured research interviews, to yield additional in-depth details for each case in the sample (González et al., 2018). This template was shared with the police officers who investigated the original case who completed this additional research interview during 2020/2021 with those missing persons who were located in a GSH (or with their relatives in those cases in which contact with the missing person was not possible) (Fyfe et al., 2015; Holmes, 2016; Sowerby & Thomas, 2017). Psychological autopsy techniques were included in the interview template conducted with relatives and friends of those missing persons who took their own life (Foy, 2006). With regard to the homicide cases, members of the research team completed an in-depth review of the cases consisting of reading police records, judicial documentation, institutional reports, and (a) completing interviews with police officers who investigated the case, (b) with the victim and perpetrator’s close family and friends, and (c) with the perpetrator in prison.

In this regard, templates for conducting the interviews as well as guidelines were adopted by both the working group and police officers. Moreover, academics and police officer members of CNDES received training in cognitive and investigation interview as well as in communication skills. Semi-structured interviews were divided into five main sections: (a) introduction and presentation, (b) relationship between interviewee and missing person, (c) lifestyle and psychosocial profile of the missing person, (d) characteristics of the disappearance, and (e) closing and ending the interview. For those cases in which judicial (sentence) and penitentiary information (psychosocial or medical reports amongst others) were available, they were also reviewed. Data processing was conducted under the requirements of Spanish data protection regulation (Organic Law 3/2018 of December 5th on Data protection and guarantee of digital rights).

### Data Matrix

The data matrix comprised 102 variables classified according to three dimensions: four variables for police codification of the case (state of the case, territorial and police demarcation and dates), 60 variables for characteristics of the missing person (sociodemographics, psychosocial, criminological, and state of health when located), and 38 variables for characteristics of the case (circumstances which surround the disappearance, motivation and typology). The nature of these variables was diverse including open text, numeric, and polytomous variables. Consequently, the dataset was refined and variables related to potential risk factors (missing person characteristics and circumstances which surround the disappearance) were recoded using a dichotomous approach based on the presence/absence (0 = no/absence; 1 = yes/presence) of the variable of interest (Almond et al., 2006; Bonny et al., 2016; Dixon et al., 2008) which is appropriate to ensure maximum clarity and reliability when using records not collected research purposes. The polytomous dependant variable (state of health when located) was also recoded into two new dichotomous variables: homicide (0 = GSH, 1 = homicide) and suicide (0 = GSH, 1 = suicide). In addition, a refinement according to the quality of the data was conducted. Pure predictors (> 80% of the observations remained in the same class) were retained and predictors with more than 50% missing values were rejected, resulting in a rejection of 13 predictors. With regard to socio-demographic factors (i.e. adults/minors, females/males, nationals/foreigners), descriptive comparison (see Table 1) showed that some sample sizes were not large enough to be analysed as isolated groups. Consequently, these were also excluded resulting in a final data matrix formed by 59 variables (see Table 2).

**Table 1:** Descriptive comparison of socio-demographic factors for homicide and suicide outcomes

		Homicide		Suicide	
		*n*	*(%)*	*n*	*(%)*
Age	Adults	32	(7.6)	42	(9.8)
	Minors	4	(0.9)	0	(0.0)
Sex	Males	15	(3.0)	34	(6.6)
	Females	21	(5.3)	8	(2.1)
Origin	Nationals	18	(2.4)	41	(5.4)
	Foreigners	18	(12.3)	1	(0.8)

**Table 2:** Variables included in the study

*Register*	*Characteristics of the missing person*	*Circumstances which surround the case*
ID of case	Family problems	Having identity/passport documents
Police force	Emotional problems	Having luggage
Territory	Work problems	Access to transport
	Delinquency problems	Having money
	Mental health issues	Having personal belongings
	Emotional problems	Having mobile
	Neurodegenerative illness	Mobile switched off
	Disability	Using mobile
	Consume/abuse of alcohol/drugs	Lack of medication
	Previous criminal conviction	Medication non-compliance
	Recent job loss	Visiting place of personal meaning
	Recent bereavement	Needs space
	Recent diagnosis of serious illness	Feeling in physical/psychological danger
	Eviction	Going to different country/region
	Insufficient income	Being on holiday
	Debts	Argument with family/friends
	Victim of crime	Family rebellion
	Busy/unstructured lifestyle	Avoiding responsibilities
	Unable to speak local language	Committing crime
	Disassociation from family	Suicide verbalisation
	Procriminal relations	Suicide note
	Previous suicide attempts	Expressing being depressed
		Being in a separation process
		Meeting partner/ex-partner
		Leaving children unsupervised
		Missing with children
		Completing large money withdrawals
		Involved in prostitution
		Being in a high-risk place
		Problems with gambling
		Visiting gambling places
		Visiting pubs
		Reached out for support
		Lack of local geographical knowledge
		Missing away from normal home
		Access to firearm
		Travelling on foot
		Being under influence of alcohol/drugs
		Missing with another person
		Extended periods of time away from home

### Data Analysis

SPSS version 21 was used for data analysis. Characteristics of the missing person and circumstances surrounding the case (see Table 2) were analysed as independent variables (predictors), with state of health when located (homicide/suicide vs. GSH) analysed as dependent variables. Firstly, bivariate descriptive analyses were conducted using contingency tables. Secondly, odds ratio (OR) was used to investigate the relationship between predictors and the missing person state of health when located. Finally, multivariate inferential analyses based on binary logistic regression (LR) were conducted to determine the statistical significance of the effects.

## Results

### Bivariate Analysis

#### Risk and Protective Factors for Homicide

When studying risk factors for homicide cases, the OR ranged from 2.21 for having an “argument with family/friends” to 17.31 for having previously been “victim of crime”. On the other hand, protective factors for homicide cases obtained an OR which ranged between 0.04 for experiencing a “family disassociation” and 0.26 for “having emotional problems” (see Table 3).

**Table 3:** Indicators associated with homicide outcomes (*n* = 887)

	Frequency (*N* = 887)					Coefficient
*Indicators*	Homicide (*n*, %)		GSH (*n*, %)		χ^2^	OR [95%, CI]
Debts	6	(11.8)	45	(88.2)	7.91**	3.54 [1.39, 9.06]
Mobile switched off	7	(3.0)	228	(97.0)	7.02**	5.23 [1.34, 20.40]
Victim of crime	16	(25.4)	47	(74.6)	90.41***	17.31 [8.07, 37.15]
Argument with family/friends	17	(6.0)	268	(94.0)	5.03*	2.21 [1.08, 4.49]
Procriminal relations	20	(6.6)	281	(96.4)	6.97**	2.54 [1.24, 5.18]
Being in a separation process	13	(23.2)	43	(76.8)	53.07***	10.23 [4.82, 21.70]
Leaving children unsupervised	6	(20.0)	24	(80.0)	20.13***	6.85 [2.61, 18.01]
Extended periods away from home	16	(20.0)	64	(80.0)	44.96***	8.22 [4.03, 16.77]
Lack of local geographical knowledge	6	(28.6)	15	(71.4)	4.72*	3.34 [1.07, 10.40]
Family problems	10	(1.8)	536	(98.2)	20.79***	0.19 [0.08, 0.42]
Busy/unstructured lifestyle	8	(1.5)	532	(98.5)	18.69***	0.19 [0.08, 0.44]
Disassociation from family	1	(0.3)	338	(99.7)	19.25***	0.04 [0.00, 0.32]
Having identify/passport documents	4	(0.9)	433	(99.1)	10.23**	0.19 [0.06, 0.59]
Travelling on foot	2	(1.0)	197	(99.0)	29.46***	0.05 [0.01, 0.22]
Emotional problems	4	(1.6)	251	(98.4)	6.98**	0.26 [0.09, 0.76]
Using mobile	2	(1.3)	147	(98.7)	8.53**	0.15 [0.03, 0.64]

The *odd ratio* coefficient (OR) indicates protection when values are inferior to 1, as long as the range does not contain the unit. Significant value (r) for risk *p < . 05, **p < . 01, ***p < .001

#### Risk and Protective Factors for Suicide

Risk factors for suicide outcomes were identified and obtained an OR range which ranged between 32.77 for existence of “suicide note” and 2.18 for “having identity/passport documents”. On the other hand, protective factors for suicide outcome cases obtained an OR which ranged between 0.14 for “procriminal relations” and 0.35 for experienced “family disassociation” (see Table 4).

**Table 4:** Indicators associated with suicide outcomes (*n* = 893)

	Frequency (*N* = 893)					Coefficient
*Indicators*	Suicide (*n*, %)		GSH (*n*, %)		χ^2^	OR [95%, CI]
Mental health issues	26	(9.1)	259	(90.9)	17.42***	3.70 [1.92, 7.10]
Neurodegenerative illness	13	(8.1)	148	(91.9)	5.52*	2.25 [1.12, 4.50]
Recent job loss	3	(15.0)	17	(85.0)	4.94*	3.83 [1.07, 13.64]
Recent bereavement	4	(20.0)	16	(80.0)	9.69**	5.14 [1.64, 16.16]
Eviction	2	(20.0)	8	(80.0)	5.11*	5.14 [1.06, 25.07]
Debts	6	(11.8)	45	(88.2)	6.59*	3.18 [1.25, 8.05]
Having identity/passport documents	30	(6.3)	433	(93.7)	4.61*	2.18 [1.05, 4.53]
Access to transport	25	(10.6)	210	(89.4)	23.23***	4.48 [2.31, 8.67]
Having money	24	(7.0)	321	(93.0)	5.32*	2.14 [1.10, 4.15]
Mobile switched off	23	(9.2)	228	(90.8)	13.40***	3.22 [1.67, 6.21]
Lack of medication	15	(8.0)	172	(92.0)	7.60**	2.54 [1.28, 5.03]
Feeling in physical/psychological danger	8	(14.3)	48	(85.7)	13.15***	4.10 [1.81, 9.66]
Expressed being depressed	20	(19.6)	82	(80.4)	67.17***	12.27 [5.88, 25.59]
Being in a separation process	9	(17.3)	43	(82.7)	19.58***	5.19 [2.32, 11.63]
Suicidal verbalisation	20	(27.8)	52	(72.2)	92.91***	14.83 [7.45, 29.52]
Suicide note	15	(51.7)	14	(48.3)	145.82***	32.77 [14.39, 74.64]
Leaving children unsupervised	5	(17.2)	24	(82.8)	10.89**	4.76 [1.71, 13.20]
Access to firearm	3	(37.5)	5	(62.5)	15.22***	11.47 [2.51, 52.311]
Criminal conviction	4	(1.4)	274	(98.6)	9.96**	0.21 [0.07, 0.61]
Family rebellion	9	(2.3)	386	(97.7)	8.95**	0.33 [0.15, 0.70]
Family disassociation	8	(2.3)	338	(97.7)	7.09**	0.35 [0.16, 0.78]
Procriminal relations	3	(1.1)	281	(98.9)	13.91***	0.14 [0.04, 0.46]
Busy/unstructured lifestyle	9	(1.7)	532	(98.3)	28.54***	0.16 [0.07, 0.34]
Reached out for support	6	(2.5)	231	(97.5)	5.98*	0.34 [0.14, 0.84]

The *odd ratio* coefficient (OR) indicates protection when values are inferior to 1, as long as the range does not contain the unit. Significant value (r) for risk *p < . 05, **p < . 01, ***p < .001

## Multivariate Analysis

Two binary logistic regressions (Wald’s backwards method) were calculated to predict homicide and suicide outcomes based on missing person characteristics and circumstances which surrounded the case.

A significant regression equation was found for homicide (*χ*^*2*^ = 19.533, *p* < 0.001), with a pseudo-R^2^ of 0.34 which was found to be low effect. The estimated coefficients for this logistic regression model imply that those missing persons who were “immersed in a separation process” and had “previously been victim of crime” were more likely to be found deceased because of homicide (see Table 5).

**Table 5:** Binary logistic regression for homicide (*n* = 887)

Predictors	*B (SE)*	Odds Ratio	95% CI [lower, upper]
Being in a separation process (1 = yes)	2.36 (0.97)*	10.66	[1.56, 72.63]
Victim of crime (1 = yes)	2.54 (0.98)**	12.79	[1.87, 87.31]
Constant	-7.19 (1.25)***		

*SE* standard error; *CI* confidence interval; significance = *** *p* < 0.001, ** *p* < 0.01, * *p* < 0.05. Model: *χ*^*2*^ = 19.533; *p* = .000; Cox and Snell *R*^*2*^ = .03; Negelkerke *R*^*2*^ = .34; Hosmer and Lemeshow *χ*^*2*^ = 0.429; *p* = .51

Another significant regression equation was found for suicide outcomes (*χ*^*2*^ = 71.513, *p* < 0.001), with a pseudo-R^2^ of 0.51 which resulted to be medium effect. The estimated coefficients for this logistic regression model imply that those missing persons who had “neurodegenerative illnesses”, “expressed being sad or depressed”, “left a suicide note”, and “did not have a busy lifestyle” were more likely to be found deceased because of a suicide (see Table 6).

**Table 6:** Binary logistic regression for suicide (*n* = 893)

Predictors	*B (SE)*	Odds ratio	95% CI [lower, upper]
Neurodegenerative illness (1 = yes)	1.44 (0.67)*	4.23	[1.13, 15.82]
Expressed being depressed (1 = yes)	1.82 (0.92)*	6.18	[1.01, 37.81]
Suicide note (1 = yes)	3.98 (1.64)**	53.92	[2.11, 1358.83]
Busy lifestyle (1 = yes)	− 1.58 (0.59)**	0.20	[0.06, 0.66]
Constant	− 1.71 (0.69)***		

*SE* standard error; *CI* confidence interval; significance = *** *p* < 0.001, ** *p* < 0.01, * *p* < 0.05. Model: *χ*^*2*^ = 71.513; *p* = .000; Cox and Snell *R*^*2*^ = .33; Negelkerke *R*^*2*^ = .51; Hosmer and Lemeshow *χ*^*2*^ = 1.903; *p* = .99

## Discussion and Conclusions

The current research explored psychosocial and criminological differences in missing person’s state of health when located, to contribute to the generation of empirical knowledge which could help to identify early indicators of missing persons at risk of suicide or homicide. The findings suggest that there are specific psychosocial and criminological factors that can influence whether an individual will be a victim of homicide or suicide when they are reported as missing, as well as high risk indicators which can provide early indication of such cases offering the potential for preventive initiatives and standardisation of first responders’ actions.

### Homicide Cases

The study of risk factors for homicide cases showed that there were some missing person characteristics and circumstances surrounding the case which could increase the probability of being a victim of a homicide: having had an argument prior to going missing, having previously been a victim of crime, having economic debts, having a mobile phone switched off, having procriminal relations, being in the process of separating, leaving children unsupervised, taking extended periods of time in a different place from the normal residence, or having a lack of local geographical knowledge. In contrast, some factors decreased the probability of being a victim of homicide (i.e. were protective factors): experiencing a family disassociation, having emotional issues, family problems, a busy lifestyle, having identity/passport documents when went missing, travelling on foot, or using a mobile phone.

These results are consistent with the small amount of existing international research on this topic. In particular, Biehal et al. (2003) and James et al. (2008) showed that being in a separation process was characteristic of the parents of those children who were victims of homicide and were reported as missing. This indicator as well as others identified in the current research (e.g. leaving children unsupervised or having previously been a victim of crime) could be characteristic of intimate partner homicides (Santos-Hermoso et al., 2022) as well as of infanticides committed in the context of intimate partner violence (Carruthers, 2016; Company et al., 2015) in which victims are reported as missing. However, internationally there is a lack of psychosocial and criminological research which allows the direct comparison of other risk factors (e.g. having procriminal relations, having previously had a previous argument, having economic debts, having a lack of local geographical knowledge, or having a mobile phone switched off). In contrast, while Biehal et al. (2003) and James et al. (2008) identified abusing alcohol was a characteristic of victims of homicide, this variable was not found to be significant when distinguishing between homicide and GSH in the context of the current research.

In the Spanish context, these findings appear consistent with previous descriptive research about missing person cases which result in homicide (García-Barceló et al., 2020a, c; Garcia-Barceló & González, 2020) in which a descriptive classification was proposed: (a) homicide outcomes related to intimate partner violence, (b) homicide outcomes related to fights and clashes, (c) homicide outcomes associated with other crimes such as sexual assaults or robberies, and (d) homicide outcomes motivated by prize or reward. The findings of this research support this proposed typology based on the identification of homicide outcome risk factors in the current research (i.e. being in a separation process, having previous arguments, having procriminal relations, a lack of local geographical knowledge, economic debts, and having previously been a victim of crime). These variables were also identified in García-Barceló and González (2020) as some of the characteristics with the highest descriptive value for each type of homicide (31.8%, 52.6%, 47%, 30%, 11.8%, and 17.3%, respectively).

In addition, comparison of these research findings with previous research on homicides which do not involve a missing person incident (Carruthers, 2016; Company et al., 2015; Santos-Hermoso et al., 2022; UNODC, 2019) shows that there are some characteristics shared by both types. Firstly, some variables identified as risk factors and descriptive of missing person cases which result in homicide in this study (e.g. being in a separation process or previously being a victim of crime) have also been identified to be characteristics of intimate partner homicides (Carruthers, 2016; Company et al., 2015; Santos-Hermoso et al., 2022). Secondly, UNODC (2019) points out that having previously had an argument or having economic debts can be trigger factors for fights/assaults which result in homicide. Lastly, UNODC (2019) has indicated that having procriminal relations is characteristic of those victims of homicides related to other criminal activities. In this manner, these similarities could support the disappearance as a specific circumstance of the homicide. Related to this, previous research shows there are some characteristics, such as “having procriminal associations”, “being in the process of separating”, and “previously having been a victim of crime”, which are especially associated with missing person cases which result in homicide (Buckley, 2012; James et al., 2008; García-Barceló et al., 2020a). Consequently, these indicators could be considered by police investigators as “red flags” for homicide.

Addressing the explanation of homicide outcomes, despite a lack of a consolidated international theoretical background and multivariate research which can explain homicide (Blackmore et al., 2005; García-Barceló et al., 2022), some descriptive approaches (Biehal et al., 2003; James et al., 2008) suggest that being in a separation process could be an important variable to consider when explaining motives and causes of homicide. The latter is partially supported by the findings of this research considering that the statistically significant variables being in a separation process and previously having been a victim of crime explained only 34% of the variance for homicide. Consequently, there is a need for additional future multivariate research focused on exploration of missing person cases which result in homicide to consolidate/extend the findings presented here.

### Suicide Cases

Suicide has been identified as one of the most prevalent causes of fatal outcomes in missing person cases (Spanish Ministry of Interior, 2021; Yong & Tzani-Pepelasis, 2020). With regard to suicide, the descriptive findings showed that those persons reported as missing who had mental health issues, neurodegenerative illness, experienced a recent job loss or bereavement, suffered eviction, had economic debts, access to transport, had money, their mobile phone was switched off, had a lack of medication, felt in physical/psychological danger, expressed being depressed, verbalised suicidal intent, left farewell/suicide notes, had passport/identity documents, left children unsupervised, or had access to firearms were at a higher risk of suicide (i.e. risk factors). However, those persons reported as missing who had procriminal relations, experienced family disassociation, had a judicial/police background, rebelled against their families, had a busy/stressful lifestyle, or reached out for support were at a lower risk of suicide (i.e. protective factors). The results of this study are consistent with international research (Sveticic et al., 2012; Woolnough et al., 2019; Yong & Tzani-Pepelasis, 2020) identifying that experiencing relationship breakdown/problems, physical health problems, financial problems, having previously attempted suicide, leaving a suicide note, or having mental health issues are key risk factors for suicide outcomes. Despite this concurrence, however, there are some important differences with the findings presented here. Firstly, this research identified that having neurodegenerative illness, experiencing a recent job loss or bereavement, being evicted, having a lack of medication, and being depressed could be interpreted as stressors or push factors (Gibb & Woolnough, 2007; Tarling & Burrows, 2004; Huey & Ferguson, 2020) of going missing which could additionally influence the perpetration of suicide. Secondly, a group of variables related to the circumstances which surround the case including having access to transport and money, taking passport/identity documents, having a mobile phone switched off, leaving children unsupervised, or having access to firearms were also identified as risk factors of a suicide outcome. Indeed, it is argued that some factors such as having a mobile phone switched off, leaving children unsupervised, or having access to firearms could be interpreted as overt signals regarding the perpetration of suicide (Azrael & Miller, 2020). Factors such as taking passport/identity documents or money could be considered a disposition of the person’s belonging prior to suicide (Park et al., 2020), while access to transport could inform about geographical displacement or the method used for suicide (Larrañaga et al., 2019; Yong & Tzani-Pepelasis, 2020).

With regard to comparing these research findings with previous research focused on studying differences between missing-suicides cases and suicides which do not involve a missing person report, characteristics such as “having mental health issues”, “having neurodegenerative illnesses”, “leaving suicide notes”, “experiencing bereavement”, and “having financial or employment problems” appear to be shared by both types and cannot distinguish between them. This supports the idea that these cases should not be addressed as clinically different by responders such as health practitioners or emergency professionals (Sveticic et al., 2012; Woolnough et al., 2019). In contrast, characteristics such as “leaving suicide notes”, “experiencing suicide threats or verbalisation”, “having economic problems”, and “debts” are specifically associated with missing person cases which result in suicide or in which the most prevalent behavioural theme is the dysfunctional one (Bonny et al., 2016; García-Barceló et al., 2020a). Consequently, when police officers assess missing person reports, these indicators could be considered “red flags” for suicide.

With regard to explanation of suicide outcomes, there is a higher amount of existing empirical research (Sveticic et al., 2012; Woolnough et al., 2019; Yong & Tzani-Pepelasis, 2020) which has studied these outcome characteristics in missing person cases in comparison to homicide cases. These studies identified that those persons experiencing relationship breakdown/problems, physical health problems, financial problems, having previously attempted suicide, leaving a suicide note, or having mental health issues were at risk of suicide (Sveticic et al., 2012; Woolnough et al., 2019; Yong & Tzani-Pepelasis, 2020). While the present research identified through multivariate techniques that 51% of the variance for suicide outcomes is explained because of having neurodegenerative illnesses, having neurodegenerative illnesses, expressing being sad or depressed, leaving a suicide note, and not having a busy lifestyle, some variables identified in the international research, such as having physical health issues, or living alone (Sveticic et al., 2012; Woolnough et al., 2019; Yong & Tzani-Pepelasis, 2020), were not recorded in the current study. The future measurement and inclusion of these variables in the multivariate models could increase the accuracy of them when trying to identify a wider explanation for suicide outcomes. Moreover, there is a further difference whereby existing international research (Sveticic et al., 2012; Woolnough et al., 2019; Yong & Tzani-Pepelasis, 2020) agrees that abusing alcohol/drugs is relevant to explaining suicide outcomes in missing person cases, but this was not found to be significant when distinguishing between homicide and suicide outcomes in the current study.

Importantly, there are some risk indicators shared by missing person cases which result in homicide and suicide: having economic debts, having a mobile phone switched off, leaving children unsupervised, or being in the process of separating. The sharing of these factors could be explained considering that some intimate partner aggressors commit homicide followed by suicide (HS; Santos-Hermoso et al., 2022) and thus could present a combination of factors such that being in a separation process relates to committing the intimate partner homicide, and then, leaving children unsupervised relates to the act of suicide. In addition, having economic debts could be interpreted both as a trigger for an argument which could result in a homicide (UNODC, 2019) and as a stressor for suicide (Sveticic et al., 2012; Woolnough et al., 2019). On the other hand, the existence of some indicators shared by homicide and suicide outcomes could link to the challenge of suspicious missing person cases in which the person is located deceased but the cause and/or reason for death is equivocal (LePard et al., 2015).

In conclusion, these findings support the notion that missing persons are a complex and multifaceted phenomenon (Alys et al., 2013). Moreover, the diverse nature of the risk and protective factors for homicide and suicide outcomes identified in the current research supports the concept that going missing and suffering harm could be the result of a combination of missing person characteristics and circumstances which surround the case (risk and protective factors) where maladaptive coping responses manifest themselves (Huey & Ferguson, 2020; Tarling & Burrows, 2004). Despite this research having generated some contribution to the characterisation and explanation of homicide and suicide outcomes, there is still a considerable need for additional multivariate analysis in this area (Blackmore et al., 2005; García-Barceló et al., 2022) to yield a much better understanding of these kinds of fatal outcomes.

### Implications for Practice

This research has generated empirical knowledge which facilitates prevention and the potential to improve the effectiveness of multi-agency response (Ferguson & Soave, 2020; Gong et al., 2017; National Crime Agency, 2016; National Crime Information Center-FBI, 2017; Spanish Ministry of Interior, 2021; Stevenson & Thomas, 2018; Todorović & Butorac, 2017). In terms of prevention, the findings could facilitate the early identification of those at risk of going missing due to becoming a victim of homicide or suicide in the future, as well as the establishment of mechanisms which could prevent its occurrence. These mechanisms could be developed and integrated in the context of individuals in contact with social services as well as educational and mental health centres. In this manner, educational and support initiatives could be adopted for those with specific risk factors. For example, where individuals are in the process of a separation, there could be potential awareness raising amongst family and friends regarding the risk of going missing and resulting in homicide or suicide, as well as to raise awareness regarding the need to maintain contact with loved ones. On the other hand, considering that indicators such as having economic debts are characteristic of both homicide and suicide outcomes, cognitive-behavioural initiatives could be provided to support those affected and at risk. Improving an individual’s understanding and ability to cope with these stressful situations could facilitate the adoption of collaborative and healthy responses to the stressor. Prevention in this manner could reduce the possibility of reigniting previous arguments, which risk a fatal outcome, or unhelpfully ruminating on problems and taking the decision to suicide. Moreover, from an applied perspective, in those cases in which practitioners from social services or mental health centres identify that a person has failed to turn up for a scheduled appointment and characteristics such as having a mobile switched off or leaving children unsupervised are identified, this could highlight to practitioners the need for early reporting to law enforcement increasing the changes of locating the person alive.

With regard to Law Enforcement Agencies’ (LEAs) response, the identification of risk factors of homicide and suicide outcomes based on psychosocial and criminological factors could facilitate the creation of support tools to improve decision-making based on an evidence-informed approach during missing person investigations. Specifically, the findings generated by this study regarding those psychosocial and criminological factors associated with homicide and suicide could serve to support the development, validation, and calibration of a structured professional judgment (SPJ) risk assessment system and decision support tool which could support law enforcement’s initial and on-going response when dealing with a missing person case. In addition, these system assessments could be linked to an evidence-based investigation protocol as well as evidence-based police resource management guide. All of this could take account of the missing person’s specific characteristics (risk indicators) associated with each assessment. Moreover, the aforementioned initiatives could be developed with the main goals of accelerating the missing person search process, increasing the possibility of locating the missing person in a good state of health and reducing the psychological impact derived from those missing person cases in which the police investigation remains active. This would facilitate the police investigation process of cases which entail high levels of complexity as well as evidencing to judicial practitioners the need for adopting specific police investigation practices (e.g. search warrants or enhanced telephony techniques) considering the proven risk factors for homicide cases. In addition, it is essential to note that the risk and protective factors for homicide and suicide outcomes identified prior to the disappearance may not take account of vulnerability and risks experienced while the person is missing. In this manner, it is necessary to consider regular reassessment of risk whenever new events occur or details are known to police officers.

### Limitations and Future Lines of Research

Although the representativeness of the sample used in this study could be ensured, the main limitation of this research was the proportion of “analytical cases” and “controls” adopted. Traditionally it has been considered valid to have an equal ratio of “cases” and “controls”, with authors (Lewallen & Courtright, 1998; Velentgas et al., 2013) indicating that adopting a ratio increases overall accuracy. However, the proportion of “cases” and “controls” adopted in the current research (36 homicide vs. 851 controls; 42 suicide vs. 851 controls) was much higher than recommended in scientific literature. In this manner, it is extremely important to highlight the need for future replication of this analysis to respect a ratio of 1:2 for “cases” “and controls”.

In addition, although regression equations were significant and allowed the identification of information which could contribute to increase the rigor of the explanations regarding homicide and suicide outcomes in missing person cases, they were found to be low and medium effect, respectively, suggesting that there is a need to conduct further research focused on the identification of other geographical and environmental factors (Newiss, 2011) which could influence going missing and resulting in a fatal outcome due to homicide or suicide. Moreover, while 51% of the variance was explained with the model generated for suicide outcomes, the percentage of explained variance for the homicide model was only 34%. This difference could be due to the fact that in the homicide cases, the person was unintentionally missing due to the action of third parties. Consequently, there is a need to study the socio-demographic, psychosocial, and criminological characteristics of the perpetrators to increase the accuracy of statistical models and explain a higher percentage of variance. In this manner and specifically regarding homicide cases, it is important to clarify that they are over-represented in the sample (specifically those related to intimate partner homicide). Based on this, future stratified samples of homicide cases should be studied to overcome this limitation. In addition, future in-depth study of the differences between homicide and suicide outcomes will facilitate the identification of variables which could differentiate between these different fatal outcomes and associated police decision-making processes during the investigation of suspicious cases. Taking an inductive profiling perspective, there is a need to identify and increase the sample of homicide cases and study the relations between characteristics of victims, perpetrators, and crime scenes through the use of multivariate statistical techniques. This will allow the construction of empirical typologies for homicide to support the decision-making process and the development of specific guidance and protocols for the investigation of homicide cases. Another challenge in this complex area relates to issues concerning the information given by the reporting person to the police. Despite research (Foy, 2016) indicating that in more than 50% of cases the reporting person suspected correctly about the reasons which motivated the disappearance, it is important to promote further research in this field since the behaviours they report may not directly be shared by the missing person, observed by a health professional, or, in indeed, the reporting person could be responsible for the disappearance.

Moreover, despite recent efforts in Spain to advance knowledge and understanding regarding missing persons, many challenges remain. The findings presented in this research specifically relate to Spanish missing person cases which result in homicide or suicide. There is a paucity of international research focused on this topic, which makes international comparisons difficult and generalisations problematic. Consequently, research which seeks to study/replicate these findings in other countries is needed. In addition, there is a need to establish further research focused on the exploration of the relationship between psychosocial and criminological factors and state of health when located in different samples of missing persons from different socio-demographic sub-groups such as sex, nationality, and age, as well as the study of psychosocial and criminological factors and missing person typologies. All of that considering previous research (Alys et al., 2013; García-Barceló et al., 2022) which indicates that socio-demographic variables, especially the age, could be considered modulating variables.

Finally, this research constitutes one of the first European (first in Spain) empirical studies using bivariate description and multivariate explanation of missing person cases which result in homicide and suicide. The findings contribute to knowledge in two main ways: (a) increasing theoretical knowledge regarding homicide and suicide in missing person cases and (b) generating applied knowledge which could facilitate the development of specific prevention strategies for homicide and suicide outcomes in missing person cases, as well as the standardisation of risk assessment procedures which could provide early risk indications for homicide or suicide to police call takers and first response teams. In this manner, contributions are made to some of the main research and operational ambitions in the field of missing persons: the identification of risk factors of harm and fatal outcomes and the establishment of effective risk assessment systems for police investigations.

## Data Availability

The datasets generated during and/or analysed during the current study belong to the Spanish Ministry of Interior but restrictions apply to the availability of these data. The data were used under license for the current study, and so are not publicly available.
